# mTOR direct interactions with Rheb-GTPase and raptor: sub-cellular localization using fluorescence lifetime imaging

**DOI:** 10.1186/1471-2121-14-3

**Published:** 2013-01-12

**Authors:** Rahul B Yadav, Pierre Burgos, Anthony W Parker, Valentina Iadevaia, Christopher G Proud, Rodger A Allen, James P O'Connell, Ananya Jeshtadi, Christopher D Stubbs, Stanley W Botchway

**Affiliations:** 1 Central Laser Facility, STFC, Rutherford Appleton Laboratory, Research Complex at Harwell, Didcot, Oxon OX110QX, UK; 2 School of Biological Sciences, University of Southampton, Southampton, SO17 1BJ, UK; 3 UCB-Pharma, 208 Bath Road, Slough, SL1 3WE, UK; 4 School of Life Sciences, Headington Campus, Oxford Brookes University, Oxford, OX3 0BP, UK

**Keywords:** FLIM, FRET, mTOR, GFP, Raptor, Rheb

## Abstract

**Background:**

The mammalian target of rapamycin (mTOR) signalling pathway has a key role in cellular regulation and several diseases. While it is thought that Rheb GTPase regulates mTOR, acting immediately upstream, while raptor is immediately downstream of mTOR, direct interactions have yet to be verified in living cells, furthermore the localisation of Rheb has been reported to have only a cytoplasmic cellular localization.

**Results:**

In this study a cytoplasmic as well as a significant sub-cellular nuclear mTOR localization was shown , utilizing green and red fluorescent protein (GFP and DsRed) fusion and highly sensitive single photon counting fluorescence lifetime imaging microscopy (FLIM) of live cells. The interaction of the mTORC1 components Rheb, mTOR and raptor, tagged with EGFP/DsRed was determined using fluorescence energy transfer-FLIM. The excited-state lifetime of EGFP-mTOR of ~2400 ps was reduced by energy transfer to ~2200 ps in the cytoplasm and to 2000 ps in the nucleus when co-expressed with DsRed-Rheb, similar results being obtained for co-expressed EGFP-mTOR and DsRed-raptor. The localization and distribution of mTOR was modified by amino acid withdrawal and re-addition but not by rapamycin.

**Conclusions:**

The results illustrate the power of GFP-technology combined with FRET-FLIM imaging in the study of the interaction of signalling components in living cells, here providing evidence for a direct physical interaction between mTOR and Rheb and between mTOR and raptor in living cells for the first time.

## Background

The mammalian target of rapamycin (mTOR) signalling pathway has a key role in cellular regulation and is involved in multiple diseases. mTOR is a central regulator of cell growth, aging, ribosome biogenesis, protein synthesis, actin-cytoskeletal organization, autophagy and metabolism. It also plays a vital role in coupling cell growth *via* signalling pathways, according to the availability of nutrients and cellular energy supplies and oxygen [1]. mTOR forms two distinct heteromeric complexes, mTORC1 and mTORC2. mTORC1 contains mTOR, raptor (regulatory associated protein of mTOR), mLST8 and PRAS40 [2-5], whilst mTORC2 contains mTOR, rictor (rapamycin-insensitive companion of mTOR), mLST8, mSin1 and protor [6-9], raptor and rictor being specific components of mTORC1 and mTORC2 respectively.

Rheb (Ras homologue enriched in brain) is a small GTP-binding protein that has been shown to promote cell growth and control cell size in mammalian cells and also in Drosophila melangaster [10], is a key protein that relays upstream signals to regulate mTORC1. The involvement of Rheb in these important complexes is still unclear. However, Rheb is reported to bind directly to the amino terminal lobe of the mTOR catalytic domain and to activate mTOR kinase in a GTP/GDP-dependent manner [11] in cell lysate studies, although a direct interaction is difficult to prove using this approach. Furthermore, evidence using the pull-down assay approach suggests Rheb associates with mLST8 and with raptor [11,12]. Both mTORC1 and mTORC2 complexes play key roles in several pathways that are involved in human cancers and in other important diseases, making the development of inhibitors of these pathways a high priority for the pharmaceutical/biotechnology industries.

It has been reported that Rheb–TSC2 GAP activity may stimulate mTOR phosphorylation and while Rheb is considered a “component” of the mTOR signalling complex, as yet there is no convincing evidence of a direct *interaction* reported between Rheb and mTOR. It is also possible that Rheb may bind to and activate mTOR-interacting proteins such as rictor, raptor or mLST8 rather than interacting with and activating mTOR directly [1].

Raptor interacts with mTOR to form a nutrient-sensitive complex that signals to the cell growth machinery [2,3]. It has also been reported that the stability of the mTOR-raptor complex increased when cells were starved of amino acids or energy generating materials [3]. However, other studies [2] obtained no evidence for changes in mTOR-raptor complex stability when cells were treated with nutrient-rich and nutrient-poor conditions. The reason for the discrepancy in the observations between these two studies [2,3] is unclear since the former report [2] failed to demonstrate an impact of the nutrient status on the stability of the mTOR–raptor complex in mammalian cells using similar experimental conditions [3,13]. Furthermore there is some evidence that raptor functions as a mTOR scaffolding protein, the binding to the TOR signalling (TOS) motif of mTOR substrates being thought to be necessary for their effective mTOR-catalyzed phosphorylation *in vivo*[14]. Hence the dynamic aspects of the interaction between mTOR, Rheb and raptor in living cells requires new approaches for the detection of interaction, such as provided by the fluorescence resonance energy transfer – fluorescence lifetime imaging (FRET-FLIM) technique for observation of appropriately labelled materials in an active environment [15-18].

Several proteins involved in the mTOR signalling pathway, including phosphoinositide 3-kinase (PI3K) [19], PDK1 [20], Akt [21], phosphatase and tensin homolog (PTEN) [22], tuberin [23], and p70S6K and its substrate S6 [24] have been found to localize in both the cytoplasm and the nucleus. In addition, mTOR, but not raptor, has been reported to shuttle between the cytoplasm and nucleus [25-27]. By contrast, a nuclear localization of Rheb has not been reported.

Fluorescence lifetime imaging utilizing confocal single and multiphoton excited state emission microscopy together with time-correlated single photon counting (TCSPC) provides an unambiguous determination of the location of fluorophores. With the discovery of green fluorescent protein (GFP) technology, significant new information on the inner workings of the living cell has been made possible through imaging approaches. The interaction of proteins in cells can be followed by utilizing steady state FRET between protein pairs tagged with appropriate GFP-fluorophores, such as enhanced GFP (EGFP, enhanced green fluorescent protein) and monomeric red fluorescent protein (DsRed). With steady state FRET the donor fluorophore, in this case GFP, is excited and would fluoresce but instead the excited state energy is re-absorbed by an acceptor fluorophore, in this case DsRed, which itself then fluoresces at a longer wavelength, provided the two fluorophores, and by inference the proteins to which they are attached, are close (within ~7 nm). Monitoring steady state FRET intensities is notoriously difficult and requires difficult corrections etc. A better method is to monitor the decreased donor lifetime which is independent of the problems associated with steady state intensity measurements and is evidence for a direct physical interaction. We have previously shown that a reduction of as little as ~200 ps in the excited state lifetime of the GFP labelled protein represents quenching through a protein-protein interaction [15-17]. In addition two-photon-excitation fluorescence lifetime imaging microscopy (2P-FRET-FLIM) analysis as used here [15-17], provides several advantages over the standard single photon method, including reduced cellular cytotoxicity of the excitation light and reduced photobleaching of the fluorophore. Greater sensitivity of the set-up is achieved through reduced sensitivity of the excitation light (>900nm) by the photomultiplier tube as a detector. Using the sensitive advanced imaging technique of time-correlated single photon counting coupled with fluorescence lifetime imaging and molecular GFP fusion technology, it was possible for the first time to probe directly the nature of the interaction between Rheb, mTOR and raptor as well as raptor and mTOR whilst providing new insights into their sub-cellular localization.

While the GFP-expression approach is excellent for providing evidence of potential interaction of proteins in living cells, since it is an over-expression, one cannot exclude the possibility that the labelled proteins may not interact at their lower natural endogenous levels. Thus there will always be some uncertainty in the approach. However, alternative methods for assessing interactions are more limiting as they all involve pull-down assays after solubilisation of the whole cell or cell fractionation which destroys localization information and itself introduces interaction artefacts as it mixes organelle contents. For these reasons the GFP approach is widely used and is the best method available for assessing potential interactions of cell components as long as one is aware of its limitations.

In this study, we report on the significant nuclear localization of Rheb together with high levels of a constant pool of mTOR (but not raptor) in HEK293, CHO and HeLa cells using advanced real-time microscopy. Interestingly, raptor was absent from the cell nucleus under all conditions investigated. A constant and direct interaction was observed between Rheb-mTOR and raptor-mTOR, but not between Rheb and raptor in the cell cytoplasm.

## Results

### Rheb localizes to both cytoplasmic and nuclear regions in mammalian cells

**Figure 1:**
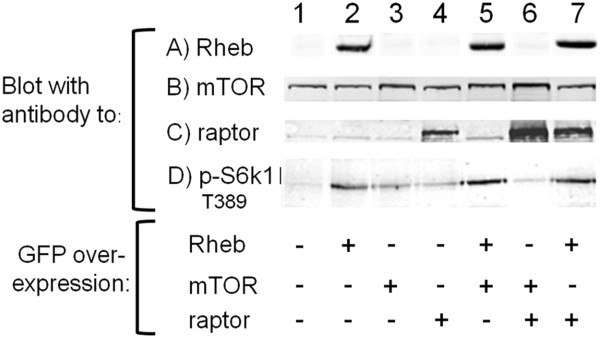
**Functionality of EGFP-Rheb, EGFP-mTOR and DsRed-raptor determined by S6-kinase activity. **EGFP-Rheb, EGFP-mTOR and DsRed-raptor were expressed in HEK cells and gels were run and blotted with the respective antibodies to Rheb, mTOR, raptor and S6-kinase (thr389). The expressed level of EGFP-Rheb is shown in Row A, lanes 2, 5 and 7 but the antibody was unable to pick up endogenous levels. The endogenous levels of mTOR can be seen in Row B, lanes 1, 2, 4 and 7 (with increased density when EGFP-mTOR is expressed (lanes 3, 5 and 6, high MW too close for large migration difference). The endogenous levels of raptor can be seen in Row C, lanes 1, 2, 3, and 5, with lanes 4, 6 and 7 showing DsRed-raptor expression.

The functionality of EGFP-Rheb was first examined by Western blots using an antibody to the phosphorylated form of S6-K1 as induced by mTORC1 signalling (see Figure 1). S6-K1-phosphorylation (Row D, lane 2) was increased due to EGFP-Rheb expression, which was therefore functional. The data indicate that it is Rheb that largely controls the level of S6-K phosphorylation. If either of the mTOR or raptor had been non-functional they would have reduced the level of S6-K phosphorylation, in fact it was increased by a small but significant amount by EGFP-mTOR and DsRed-raptor expression. Therefore EGFP-mTOR and DsRed-raptor were likely also functional.

**Figure 2:**
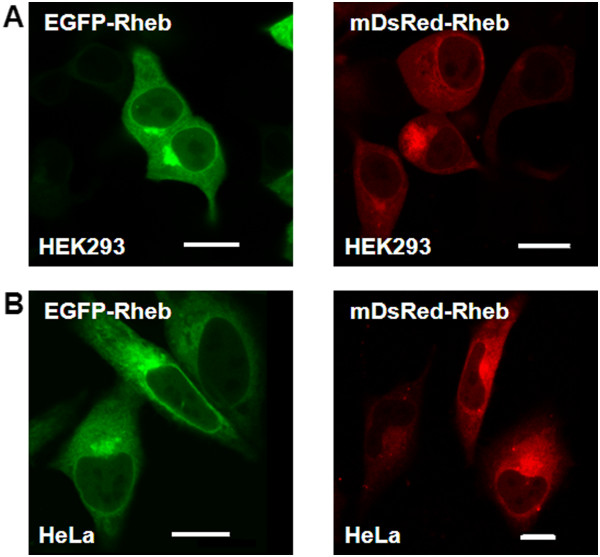
**EGFP**-**Rheb and DsRed**-**Rheb expression in mammalian cells. A**) HEK293 cells and **B**) HeLa cells were transiently transfected with EGFP-Rheb (left panel) and DsRed-Rheb vector (right panel). 24h following transfection, and the live cells analyzed under a Nikon TE2000 U confocal microscope. Bar 8 μm.

EGFP and DsRed tagged proteins were observed to be efficiently expressed in several mammalian cell lines under investigation. Figure 2 shows confocal images of the transiently expressed fluorescent proteins fused with Rheb protein in HEK293 and HeLa cells. The transfection expression levels for the cell lines used in this study differed somewhat, in that HEK293 cells showed more than 80% transfection efficiency, compared to CHO and HeLa cells at ~60% (CHO image data not shown), likely due to differences in the optimal conditions for transfection for the three cell lines. Fluorescently tagged Rheb, raptor and mTOR were confirmed by SDS-PAGE and gel electrophoresis in that the correct fluorescent protein was tagged to mTOR, Rheb and raptor and that they were constructed and expressed correctly (data not shown). The main localization of Rheb and mTOR appeared to be the perinuclear regions in the Golgi and ER.

**Figure 3:**
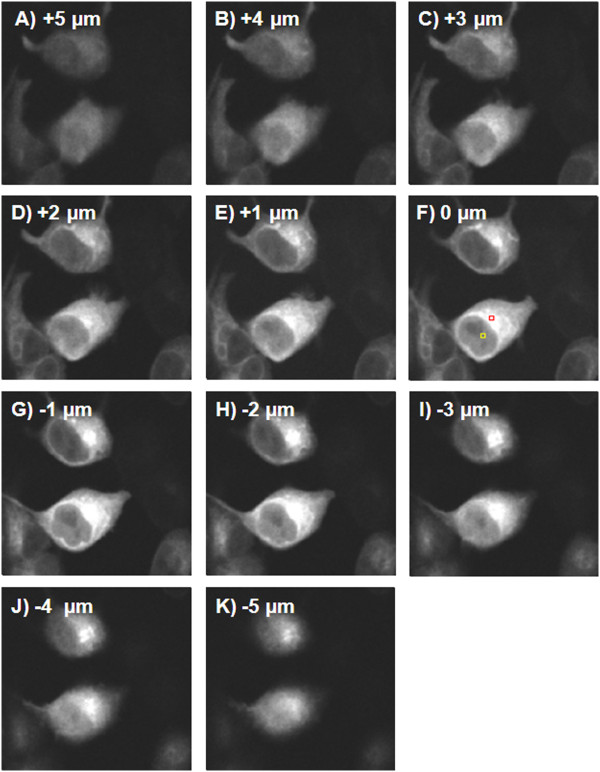
**3D Stack images of multiphoton-induced fluorescence of HEK293 expressing EGFP-Rheb.** Fluorescence from TCSPC images of live HEK293 cells was acquired using multiphoton excitation (920 nm laser excitation, 520 nm emission) following 24h transfection. Raw data presented without further image processing. Images show clear Rheb nuclear localization. Yellow and red boxes (F) refer to nuclear and ER/Golgi regions taken for comparative photon count. Image size 120x100 μm.

**Figure 4:**
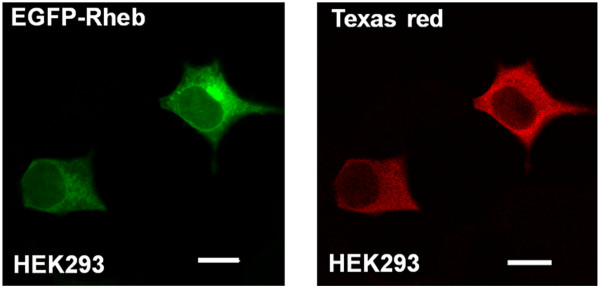
**Immunohistochemical staining of Rheb. **HEK293 cells expressing EGFP-Rheb (LEFT PANEL) or cells fixed in 4% formaldehyde and treated with anti-Rheb antibody in conjugation with Texas red-labelled secondary antibody (RIGHT PANEL) were imaged with confocal microscopy. Bar 10 μm.

**Figure 5:**
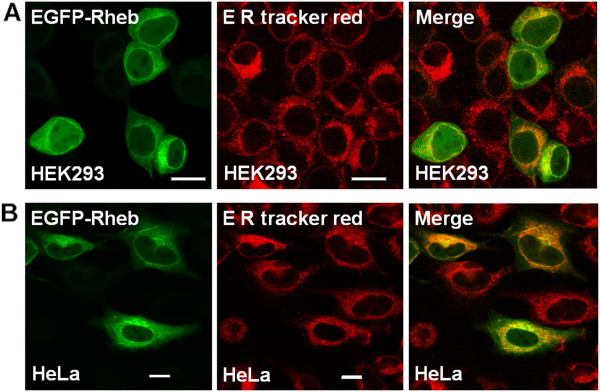
**Sub**-**cellular localization of Rheb on ER. A**) HEK293 cells; **B**) HeLa cells were transiently transfected with EGFP-Rheb. Twenty-four hours following transfection cells were stained with 1 μM ER Tracker red and live cells analyzed by confocal microscopy. The images reveal that Rheb localizes to the ER Bar 8 μm.

**Figure 6:**
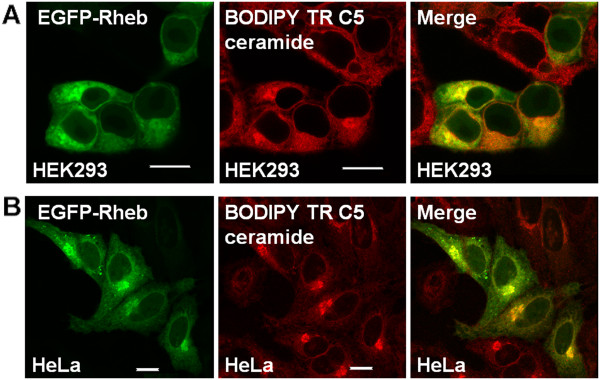
**Sub-cellular localization of Rheb on Golgi. A) **HEK293 cells; **B) **HeLa cells were transiently transfected with EGFP-Rheb. Twenty-four hours after transfection cells were stained with 5 μM BODIPY TR C5 ceramide and live cells analyzed by confocal microscopy. The images reveal that Rheb localizes to the Golgi. Bar 8 μm.

To determine if EGFP-Rheb was present within the cell nucleus, we obtained a 3D-multiphoton TCSPC image Z-stack of HEK293 cells expressing the fluorescent protein (Figure 3A-K). Using this technique, it was possible to observe the presence of EGFP-Rheb protein throughout the cell with significant expression levels within both cytoplasmic and nuclear regions. Taking into account the average fluorescence intensity of EGFP-Rheb within the cytoplasmic/Golgi regions (52000 photon counts per second, Figure 3F red box), compared to that within the nuclear region (26000 photon counts per second, Figure 3F yellow box), we can estimate Rheb expression levels of ~40% within the cell nucleus and ~60% within the cytoplasm, the Golgi apparatus and ER together. In HEK293 cells expressing EGFP-Rheb a cytoplasmic and nuclear localization was found (Figure 4). In addition we performed immunohistofluorescent staining using an anti-Rheb antibody (Figure 4), although, as is well known, Rheb antibodies are of somewhat low quality, perinuclear and nuclear staining is apparent. Co-localization studies using a Golgi fluorescent probe (BODIPY Texas Red ceramide, Invitrogen) and a probe for the endoplasmic reticulum (ER-Tracker Red; glibenclamide BODIPY TR, Invitrogen) revealed that EGFP-Rheb localized to Golgi and ER (see Figures 5, 6).

### mTOR shows some nuclear but mainly ER and Golgi localization in mammalian cells

**Figure 7:**
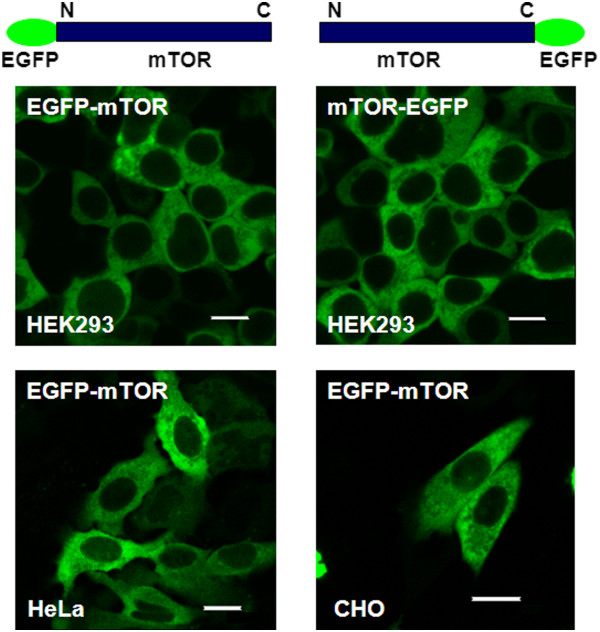
**EGFP-mTOR expresses predominantly in the cytoplasm of mammalian cells. **EGFP tagged mTOR expression was studied in HEK293, HeLa and CHO cells. The cells were transiently transfected with EGFP tagged mTOR and expression was analyzed in live cells following 48h of transfection using a Nikon TE2000 U inverted microscope and EC1 confocal system. (EGFP-mTOR; EGFP tagged at the N-terminal of mTOR and mTOR-EGFP; EGFP tagged at the C-terminal of mTOR). Bar 8 μm.

The localization of mTOR in HEK293, HeLa and CHO cells was studied by transient transfection with EGFP-tagged at either end of mTOR. Cells were examined for expression following 24h and 48h of transfection. HEK293 and HeLa cells showed mTOR expression mainly in the cytoplasm with some detectable but weak presence within the cell nucleus as shown in Figure 7.

Using the TCSPC technique, we observed the average percentage count in three independent experiments to be ~20% EGFP-mTOR in the nucleus and ~80% within cytoplasmic regions of HEK293 cells (error of ± 6%, SD in both regions) (data not shown) (note that this quantification information is lost within standard confocal laser scanning microscopy images). The maximum nuclear expression of EGFP-mTOR observed in populations of HeLa cell was ~32% (± 8%) while in CHO cells the number was ~26% (± 6% in both regions) (data not shown).

### Raptor expression is observed only in the cytoplasm

**Figure 8:**
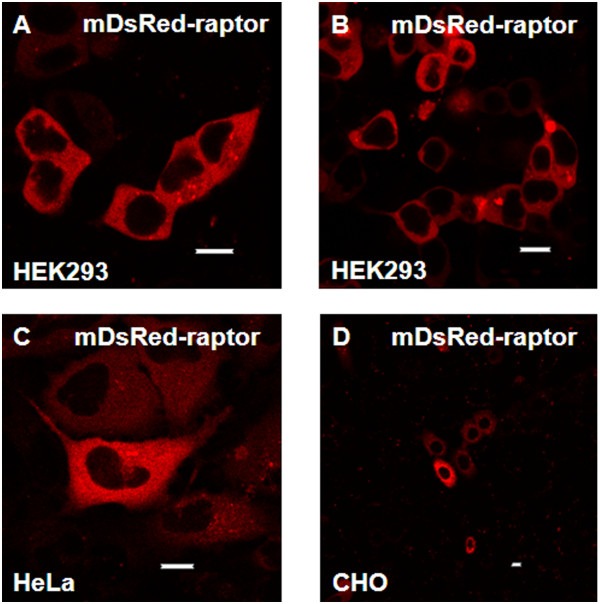
**Sub-cellular localization of transiently transfected DsRed-raptor in different mammalian cell types. **DsRed tagged raptor was expressed in HEK293 cells for **A) **24h and **B) **48h, HeLa cells for 48h, **C) **and CHO cells for 48h, **D)**. Live cells were analyzed for various times following transfection using a Nikon TE2000 U confocal microscope. Bar 8 μm.

The localization of raptor was studied by transiently transfecting HEK293, HeLa and CHO cells with a vector for DsRed-raptor. Figures 8A, B show that in HEK293 cells DsRed-raptor was predominantly within the cytoplasm, at 24h and 48h after transfection in punctate structures. Transient transfection of DsRed-raptor in HeLa (Figure 8C) and CHO cells (Figure 8D) showed a similar pattern of expression.

### EGFP-mTOR shows a direct interaction with DsRed-Rheb that is unaffected by rapamycin

**Figure 9:**
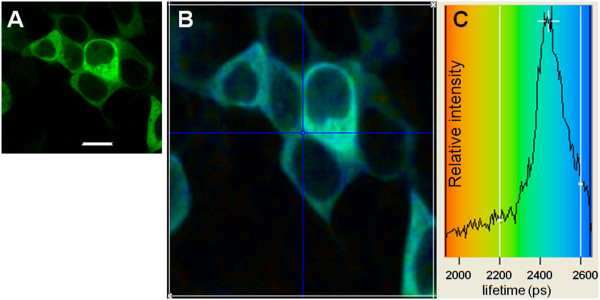
**Fluorescence lifetime imaging of EGFP-mTOR expressed in HEK293 cells. A) **Confocal image of EGFP-mTOR expressed in HEK293 cells following 48 h of transfection; **B)** Lifetime image of the same cells (colour coding for the lifetimes is shown in (**C**)), image size 120 x 110 μm; **C) **Lifetime distribution taken for the entire area (white border) shown in **(B)**. The data collection time for lifetime images were optimally 3 accumulations of 30 sec. with an image dimension of 128×128 pixels (the blue cross lines are for single pixel lifetime values which were not used). A fluorescence lifetime value was determined from the distribution centre in** (C)**, representing a value for the entire area shown in **(B) **and was ~2450 ± 100 ps, taken from three independent experiments.

To investigate mTOR binding-partner interactions, transiently expressing EGFP-mTOR in HEK293 and HeLa cells were first obtained and analyzed using FLIM, to obtain the control excited state lifetime (donor chromophore) 48h after transfection (Figure 9). A lifetime image of HEK293 cells transfected with EGFP-mTOR for 48h is shown in Figure 9, with the corresponding FLIM image. In three independent experiments the lifetime of EGFP-mTOR averaged for all of the cells was found to be 2400 ± 100 ps. EGFP-mTOR located within the nucleus showed a similar lifetime to that in the cytoplasm.

**Figure 10:**
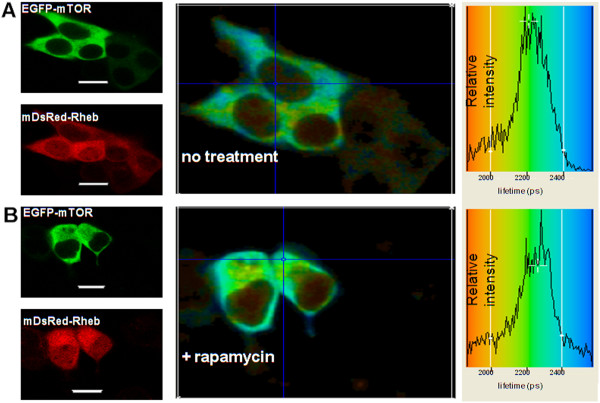
**DsRed-Rheb direct interaction with EGFP-mTOR in HEK293 cells and lack of effect of rapamycin. (A, B) **LEFT PANELS: Confocal 488 nm excitation images of EGFP-mTOR (co-expressed with DsRed-Rheb, after 48 h of transfection) and 543 nm excitation images of DsRed-Rheb, without **(A) **and with **(B) **rapamycin (100 nM) treatment for 24 h. **(A, B) **MIDDLE PANELS: Lifetime images of the co-transfected cells (colour coding for the lifetimes is shown in the RIGHT PANELS). **(A, B) **RIGHT PANELS The lifetime distributions taken for the entire area (white border) as shown in the MIDDLE PANELS. The lifetime value from the data in **(A) **RIGHT PANEL for the EGFP (attached to mTOR) is reduced due by quenching by DsRed (attached to Rheb) from ~2450 ± 100 ps for EGFP alone (see Figure 9) to 2200 ± 100 ps (n=3) here, thus showing a direct interaction. The data in **(B) **RIGHT PANEL for the lifetime of the EGFP (attached to mTOR), after treatment with rapamycin there was no relief of the quenching indicating it does not impact on the direct interaction. The data collection time for lifetime images was optimally 3 accumulations of 30 sec. with an image dimension of 128×128 pixels, bar 8 μm.

HEK293 cells co-expressing EGFP-mTOR with DsRed-Rheb over a 48h of transfection is shown in Figure 10A. The average excited state lifetime of the EGFP-mTOR in the presence of DsRed-Rheb was determined to be 2200 ± 50 ps in three independent experiments, the reduced level due to a direct interaction between Rheb and mTOR, due to energy transfer from EGFP to DsRed. In HeLa cells, a similar quenching in the lifetime of EGFP-mTOR (2100 ± 100 ps) was observed in co-transfected cells again showing a direct interaction and evidence for a direct mTOR/Rheb interaction in HEK293 cells was observable whether the EGFP was at the N- or C- termini of mTOR (data not shown).

Analysis of the average lifetime of the EGFP-mTOR from the nuclear regions in HEK293 cells was determined to be ~2000 ± 100 ps in three independent experiments. HeLa cells showed similar expression in the nuclear regions of EGFP-mTOR and DsRed-Rheb co-transfected cells (data not shown). Quenching in the lifetime of the donor (EGFP-mTOR) from 2400 ± 100 ps to 2000 ± 100 ps in the nuclear regions indicates that EGFP-mTOR and DsRed-Rheb interact (due to excited state energy transfer) differently (i.e. they are closer) in the cell nucleus than in the cytoplasm, likely brought about by a different conformation of the mTOR/Rheb complex. Importantly, in this study EGFP-mTOR and DsRed-Rheb were consistently observed to be expressed at similar levels in HEK293 cells (~40%), whilst these levels were different when expressed alone (~30% for mTOR alone but ~40% for cells also expressing Rheb).

The effect of rapamycin on the direct interaction of EGFP-mTOR and DsRed-Rheb expressed in HEK293 cells was determined by a 24 h treatment (100 nM). The EGFP lifetime (2200 ± 100 ps, data of three independent experiments) was unaffected by the treatment showing rapamycin had no impact on the interaction (see Figure 10B).

Although the interaction of Rheb with mTOR has been widely inferred by indirect approaches this is the first live cell imaging work showing the interaction occurs and does so in specific regions of the cell. Although it suffers from the disadvantage that we are looking at over-expression it enables us to examine the mechanisms and consequences of the interaction in a living cell.

### Raptor interacts directly with mTOR but not with Rheb and the mTOR-raptor interaction is unaffected by rapamycin

**Figure 11:**
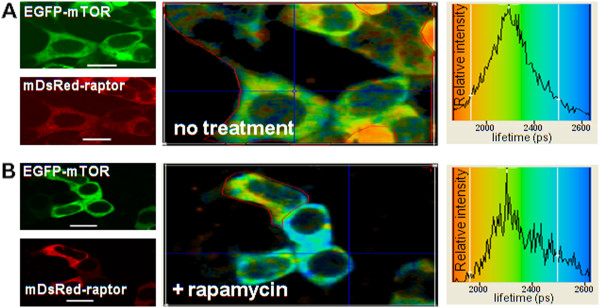
**EGFP-mTOR direct interaction with DsRed-raptor in HEK293 cells and lack of effect of rapamycin. (A, B) **LEFT PANELS: Confocal 488 nm excitation images of EGFP-mTOR (co-expressed with DsRed-raptor, after 48 h of transfection) and 543 nm excitation images of DsRed-raptor, without **(A) **and with **(B) **rapamycin (100 nM) treatment for 24 h. **(A, B) **MIDDLE PANELS: Lifetime images of the co-transfected cells (colour coding for the lifetimes is shown in the RIGHT PANELS). **(A, B)** RIGHT PANELS The lifetime distributions taken for the the region taken here is within the area of the thin red line (not the entire area as previous Figures) as shown in the MIDDLE PANELS. The lifetime value from the data in **(A) **RIGHT PANEL for the EGFP (attached to mTOR) is reduced due to quenching by DsRed (attached to raptor) from ~2450 ± 100 ps for EGFP alone (see Figure 9) to 2300 ± 100 ps (n=3) here, thus showing a direct interaction. The data in **(B) **RIGHT PANEL for the lifetime of the EGFP (attached to mTOR) for the cell demarked by the thin red line (the cell below being omitted as it is not expressing DsRed-raptor, see LEFT PANEL), after treatment with rapamycin there was no effect on the lifetime centre (2300 ps ± 100 ps). The data collection time for lifetime images was optimally 3 accumulations of 30 sec. with an image dimension of 128×128 pixels, bar 8 μm.

EGFP was tagged to either end of mTOR and DsRed was tagged to the N-terminal of raptor and lifetimes measurements of GFP observed. The excited state lifetime of the donor EGFP-mTOR was determined in HEK293 cells co-expressing DsRed-raptor. The average lifetime of the whole area of the image was 2300 ± 100 ps (Figure 11A). The corresponding confocal images of the co-expressed cells excited separately at 488 nm for EGFP-mTOR and 543 nm for DsRed-raptor, respectively, as shown in Figure 11A, shows high expression levels (>70% of cells) of both proteins. The quenched lifetime of the donor fluorophore in co-transfected cells suggests that the EGFP-mTOR and DsRed-raptor directly interact under normal growth conditions within the perinuclear regions, as indicated by Golgi and ER confocal co-localization data. Similar results were also observed in HeLa and CHO cells (data not shown). Analysis of cells co-expressing EGFP-Rheb (donor) and DsRed-Raptor (acceptor) in HEK293 and HeLa cells gave an unquenched excited state average lifetime of the donor of 2400 ± 100 ps indicating a lack of a direct interaction between Rheb and raptor (data not shown).

HEK293 cells were co-transfected with EGFP-mTOR and DsRed-raptor, and 24h later were treated with rapamycin at 2h or 24h. There was no effect on the EGFP-mTOR (quenched) excited state lifetime (~2300 ± 100 ps) indicating that the interaction was not affected by rapamycin treatment (Figure 11B). Although there may be marginal unquenched EGFP (shoulder at 2400–2500 ps, Figure 11B RIGHT PANEL), however, because the mTOR-raptor interaction is not as close as Rheb-mTOR (quenched EGFP lifetime 2300 ps vs 2200 ps, respectively) it is difficult to be certain if this indicates marginal effects of Rapamycin.

### Amino acid starvation modifies the localization and the nature of the direct interaction of mTOR with Rheb

**Figure 12:**
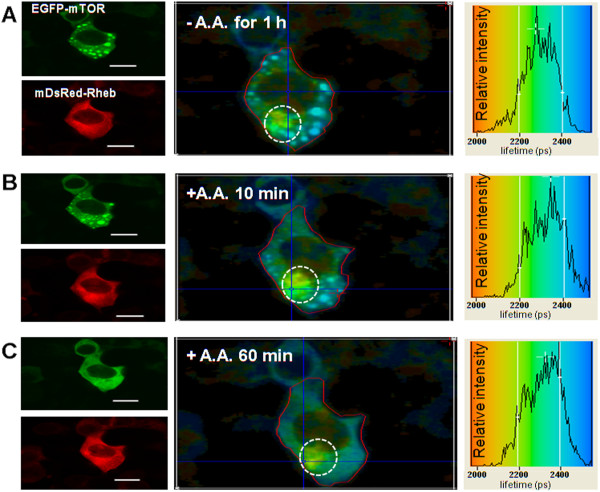
**Amino acid starvation and re-stimulation does not affect the direct interaction between EGFP-mTOR and DsRed-Rheb in perinuclear regions. **(**A-C**) LEFT PANELS: Confocal 488 nm excitation images of EGFP-mTOR (co-expressed with DsRed-Rheb, after 48 h of transfection) and 543 nm excitation images of DsRed-Rheb, for HEK293 cells serum-starved overnight and then amino acid starved in D-PBS for 1 h (**A**) following which the amino acids were added back **(B, C)**. Punctate structures appear after amino acid removal and disappear after adding back the amino acid and appear not to contain Rheb. **A**-**C**) MIDDLE PANELS, Lifetime images of the co-transfected cells (colour coding for the lifetimes is shown in the RIGHT PANELS). **(A-C) **RIGHT PANELS The lifetime distributions taken for the cell area (within red line) as shown in the MIDDLE PANELS. A, revealing *both *quenched and unquenched EGFP reflecting the mixture of perinuclear mTOR/Rheb and punctate mTOR), as reflected in the lifetime image; **B) **the same cells after 10 min of amino acid re-stimulation; **C) **after 60 minutes of amino acid re-stimulation when the punctate structures had disappeared. At all time-points the area marked by the white circle, mainly ER/Golgi, shows a quenched lifetime i.e. Rheb interacting with mTOR. Bar 8 μm.

Under conditions of amino acid starvation, punctate structures or granules were observed for EGFP-mTOR (Figure 12A). When amino acids were added back the punctuate structures gradually diminished and had disappeared by ~60 mins (see Figure 12C), or much more rapidly after addition of serum (FCS) alone (data not shown). Also amino acid starvation and re-stimulation in HEK293 cells led to a distinct region of interaction, indicated by different levels of EGFP-lifetime quenching, to ~2100 ps in the perinuclear region and to ~2400 ps in the punctuate structures. Again the results indicate that Rheb and mTOR may interact differently in the two regions. It is important to note that the EGFP-Rheb subcellular localization and distribution is unaffected by this treatment

### Amino acid starvation does not affect the localization or the direct interaction of mTOR with raptor

**Figure 13:**
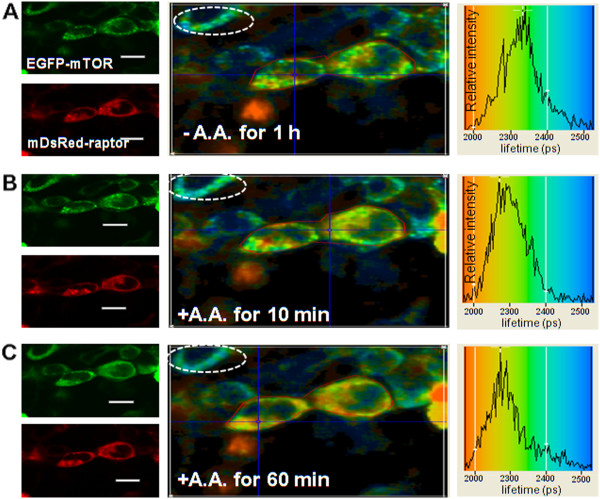
**Amino acid starvation and re-stimulation effect on the direct interaction between EGFP-mTOR and DsRed-raptor. **(**A**-**C**) LEFT PANELS: Confocal 488 nm excitation images of EGFP-mTOR (co-expressed with DsRed-raptor, after 48 h of transfection) and 543 nm excitation images of DsRed-raptor, for HEK293 cells serum-starved overnight and then amino acid starved in D-PBS for 1 h (**A**) following which the amino acids were added back (**B**, **C**). Punctate structures appear after amino acid removal and disappear after adding back the amino acid. (**A**-**C**) MIDDLE PANELS, Lifetime images of the co-transfected cells. The colour coding for the lifetime is shown in the right panel which is a lifetime distribution of the area of EGFP-mTOR and DsRed-raptor co-expressing cells (area marked by red line in the middle panel) (**A**-**C**) RIGHT PANELS The lifetime distributions taken for the cell area (within red line) as shown in the MIDDLE PANELS. A, revealing quenched EGFP reflecting a direct interaction of mTOR/raptor both in the punctate structures and other regions. Note that the lifetime of EGFP mTOR is slightly reduced, to about 2350 ps, possibly indicating that in the punctate structures there may still be some mTOR-raptor interaction but 'looser' under amino acid starvation conditions (compare Figure 11). (A cells with only EGFP-mTOR expression is shown for comparison, white circle). After amino acid re-stimulation (**B**, **C**) the punctate structures disappear and the quenched lifetime for the EGFP returns to <2300 PS.

The mTOR-raptor interaction was also observed in small punctate-like structures in the cytoplasm as for Rheb in the perinuclear region, under amino acid starvation conditions (Figure 13). Under conditions of amino acid starvation and subsequent re-addition showed no significant change in the EGFP-mTOR lifetime (2200 ± 100 ps) (Figure 13) and therefore we conclude the interaction between EGFP-mTOR and DsRed-raptor remains unchanged. Also the localization and distribution of DsRed-raptor is unaffected by the amino acid starvation treatment.

## Discussion

This study is the first to show a direct interaction between Rheb and mTOR in specific regions of living cells. Although previous studies have suggested an interaction using pull-down approaches [11], the ability to be able to study localization and interaction on a (live) cell-by-cell basis is important when studying a signalling complex which may play differing roles simultaneously in different parts of the cell, as appears to be the case with the mTORC1 complex. The FRET-FLIM approach offered two other advantages. It was able to show a direct interaction of both Rheb and raptor with mTOR, also different conformations of the interacting components could be distinguished by this technique, such information being unavailable using a pull-down assay approach.

Both EGFP- and DsRed-tagged Rheb appeared to locate in perinuclear regions in the Golgi and ER, with no difference in the expression levels in transfected cells. Previous studies of GFP-tagged Rheb have also shown such perinuclear accumulation [28-30]. The insensitive nature of confocal image acquisition (i.e., because of analogue signal thresholding) can make it difficult to set the threshold of standard confocal photomultiplier tubes without losing weaker signals. Consequently, gain and background levels can lead to either signal loss or oversampling. The use of highly sensitive time-correlated single photon counting, such as TCSPC, allows fluorescence photons to be detected above any background level as well as the ability to quantify the number of photons detected. Thus, in this current study using TCSPC, we report persistent and observable EGFP-Rheb presence within both cytoplasmic and also the sub-cellular nuclear regions, although the signal from the nucleus is somewhat weak and it may possibly be due to the fact that it is over-expressed. However, regarding a nuclear Rheb presence it is important to note (i) it has been suggested that mTOR shuttles between the nuclear region and cytoplasm [25-27], and (ii) that mTORC1 plays a key role in ribosome biogenesis, a process which occurs in the nucleolus [1]. The presence of Rheb within the cellular nucleus suggests it might interact with mTOR in that compartment. Although the role for Rheb within the cell nucleus is unclear and whether it simply resides there or can indeed shuttle in and out is not known, however, it is likely to have a role in directing mTOR localization and therefore downstream signalling within the nucleus. The interaction between Rheb and mTOR observed is unlikely to be due to mis-localization since randomly distributed protein molecules will be sufficiently far apart (>>10 nm) to limit direct stable interaction.

It has also been reported by using timed imaging of live cells that, following brief association with ER, EGFP-Rheb localizes to highly-ordered distinct structures within the cytoplasm that display the characteristics of Golgi membranes [28]. Rheb was also reported to be localized with mitochondria [31], along with FKBP38 and mTOR [32]. It has also been shown that GFP-tagged Rheb co-localizes with Rab7, a marker for endosomal and lysosomal structures [33]. Our studies did not reveal a co-localization of EGFP-Rheb with Mitotracker (data not shown) in keeping with observations that ectopically expressed Rheb does not localize to mitochondria [30]. Previously it has been reported that EGFP-tagged Rheb showed a granule-like fluorescence pattern in the cytoplasm, while not being found at the plasma membrane or in the nucleus, and that it has mainly an endomembrane localization [34]. However, several upstream regulators of mTOR have been reported to be expressed in the nucleus as well as in the cytoplasm and mTOR plays an important role in the regulation of translation. Evidence for a nuclear localization of mTOR has also been provided previously based on data from immunofluorescence in conjunction with cell fractionation and Western blot analysis [25-27].

Other components of the pathway such as tuberin, Rheb and p70S6K also localize to the cytoplasm (see Introduction). Indeed, tuberin, an upstream regulator of Rheb has been reported to localize in the cytoplasm and nucleus [23], similar to p70S6K, one of the major substrates of mTORC1 [24]. In addition, mTOR was also found to shuttle between the cytoplasm and the nucleus, this being required to regulate the mitogenic stimulation of p70S6K activation and 4E-BP1 phosphorylation [25]. This study provides supporting evidence for the nuclear localization of Rheb for the first time as well as mTOR in live cells. The nuclear localization of mTOR is likely an evolutionarily conserved phenomenon and may play an essential role in mTOR signalling and functioning as previously suggested [35]. With regard to raptor, previous studies using immunofluorescence have shown that raptor is localized within cytoplasm [33]. The live cell confocal imaging here also showed a cytoplasmic localization and absence from the nucleus. Furthermore lifetime data revealed that raptor interacts with mTOR both in the cytoplasm and also within the amino-acid starvation-induced punctate structures within the cytoplasm (see below).

The involvement of Rheb in the mTORC1 complex has remained an important point of interest since it was shown to interact with mTOR due to the potential of its ability to control activity of the complex through its GDP/GTP bound state [11,12,36]. Rheb is reported to bind directly to the amino terminal lobe of the mTOR catalytic domain and to activate mTOR kinase in a GTP/GDP-dependent manner in cell lysate studies. Furthermore, using a pull-down assay, Rheb has also been shown to associate with mLST8 and with raptor [11,12].

One of the key goals of this study was to investigate whether Rheb and mTOR *directly* interact in living cells and whether this interaction is affected by conditions where mTORC1 signalling is impaired (implemented *via* nutrient starvation or rapamycin treatment). The immunoprecipitation/cell lysate methods previously used are susceptible to artifacts due to the lysis conditions used and do not distinguish between direct and indirect interactions. Here, we were able to demonstrate a direct interaction of DsRed-Rheb with EGFP-mTOR (irrespective of whether the DsRed was bound to the C- or N- termini of Rheb. A direct DsRed-raptor interaction with EGFP-mTOR was also shown. By contrast, the lifetime of EGFP of EGFP-Rheb was not reduced when co-expressed with DsRed-raptor (results not shown), consistent with them not interacting directly, however, considering the large size of mTOR (280 kDa) compared to those of Rheb (21 kDa) and raptor (150 kDa), it is possible that their positions on the mTOR are further apart than the distance for efficient FRET (~7 nm). Therefore the results are consistent with a model where the signal must pass from Rheb *via* mTOR to raptor and on to downstream kinases. From the recent cryo-electron microscopy structure the N-terminus of mTOR would appear to interact with the flat face of a single raptor molecule forming one interface [37], while C-terminal of mTOR interacts with the side of a second raptor molecule forming a second interface. The interaction data from the present live cell study are consistent with the proposed model of this structural study where there may be more than one raptor molecule binding to mTOR.

**Figure 14:**
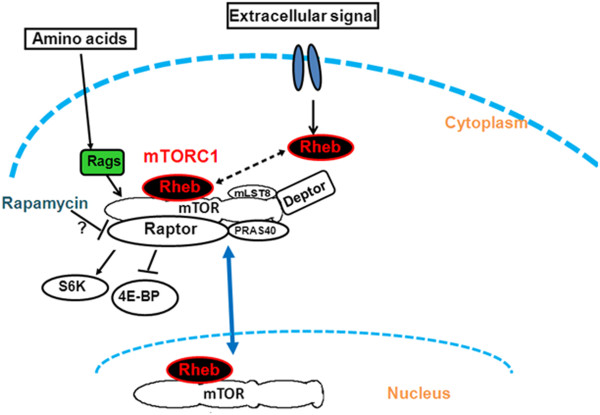
**Rheb expression and translocation into the nucleus. **Rheb activation by diverse extracellular signal enables Rheb to directly interact with mTOR. Rheb possibly binds to mTOR to shuttle between nucleus and cytoplasm or it may translocate alone to the nucleus for as yet unknown function (scheme modified from [42].

There was evidence from different levels of EGFP-mTOR lifetime quenching in the cytoplasm and the nucleus by DsRed-Rheb that the nature of the direct interaction in these two regions may differ, furthermore, the finding of significant Rheb in the nucleus is a new finding and suggests an increased levels of mTOR also within the cell nucleus. The mechanism for the increased nuclear concentration and details of the nature of the interaction remain to be investigated. From these studies, a revised Rheb binding to mTOR is proposed to include Rheb nuclear localization (Figure 14). It is clear the Rheb localization in the nucleus has important implications for mTOR signalling.

There is ongoing interest in the mechanism of action of the antagonistic action of rapamycin on mTOR signalling, not least due to its anti-cell growth/cancer drug potential. Using the GFP/FRET-FLIM approach the addition of rapamycin was not seen to affect the localization of either mTOR, Rheb or raptor, nor their interaction. Also, in agreement with other studies [38], neither amino acid starvation nor rapamycin treatment had an influence on mTOR-Rheb interactions. Regarding the effect of rapamycin on mTOR-raptor interactions, Oshiro and co-workers [39] reported that rapamycin dissociates the interaction while others [2] did not observe dissociation after rapamycin treatment in agreement with a bead-pull-down assay [7] and our results. Importantly, our data from living cells imply that rapamycin works by a mechanism other than by a complete dissociation of the mTOR-raptor interactions. Rapamycin may act by other mechanisms, as has been suggested [14], or by directly acting on the catalytic activity of mTORC1 [40]. Our observation is also in keeping with recent work [40] suggesting that the binding of rapamycin-FKBP12 to the FRB domain of mTOR induces an allosteric conformational change that reduces the intrinsic catalytic activity of mTORC1. Additional work is still required to understand how rapamycin inhibits some of the functions of mTORC1.

The punctate localization of mTOR is brought about by amino acid withdrawal, after re-addition of amino acids the punctate structures dissipate (at least as visualized by EGFP-mTOR). While Rheb did not associate with these structures, there was heterogeneity in the raptor distribution as it seemed to co-associate with mTOR in some but not all cases within a single cell. This being an over-expression and the lifetime analysis being averaged over an entire cell makes interpretation difficult in terms of what would be occurring in the endogenous situation. However, it does seem that under conditions of amino acid withdrawal, the mTOR-raptor association is looser (inactive?). This is seen as a quenching (revealed as reduced mTOR lifetime) of the EGFP (attached to mTOR) by the DsRed (attached to raptor) under amino acid withdrawal conditions being *less* than in the amino acid presence. mTOR activity is regulated by amino acid availability *via* a “ragulator” complex (includes RAG GTP'ases) directly associating with mTOR, as recently described [41], where punctate structures were also observed. However, in that study it was shown that in the absence of amino acids, mTOR is found in punctate structures in cells, but then concentrates in larger structures (lysosomes) after amino acid addition, whereas we are showing mTOR in the punctate structures as a result of amino acid starvation. It is possible that over-expression caused our result or it could be some other difference.

## Conclusions

In summary, Rheb, mTOR and raptor mainly reside on Golgi/ER like structures, where mTOR directly interacts with both Rheb and raptor, shown here for the first time in living cells using FRET-FLIM methodology. A clear localization of Rheb within the mammalian nucleus was also shown (scheme 1). Rapamycin did not affect the elevated levels of mTOR-Rheb or mTOR-raptor interactions, implying that rapamycin does not inhibit mTORC1 signalling by disrupting this complex. Amino acid starvation resulted in formation of complexes (containing EGFP-mTOR) that appear as punctuate structures which dissipate after re-addition of amino acids or serum. The interaction of raptor-mTOR was not affected by the lack of amino acids whilst the Rheb-mTOR association was 'loosened' within the granules but not in the perinuclear regions. Although GFP-technology is very widely used, one needs to be aware of the disadvantage in that the method uses over-expression of the tagged protein which may impact on the interactions and downstream target activities. For this reason caution is required in interpretations and minimally effects on these downstream activities needs to be checked to confirm functionality, as we have done here using S6-kinase activities. Overall we demonstrate that the advanced time-resolved FRET-FLIM technology provides a powerful protocol to investigate signalling pathways and it highlights the physical capabilities of the technique to provide much needed information for developing and testing drugs designed to target specific pathways in real-time in living cells.

## Methods

### Materials and Cell culture

The cDNA for Rheb was from Gene Service, raptor from Addgene and mTOR from Origene. Cellular markers for Golgi, ER and mitochondria were obtained from Molecular Probes, Invitrogen. Rapamycin was purchased from Sigma.

HEK293 and HeLa cell lines were kindly provided by Dr. Breda Twomey (UCB Pharma, UK) and the Chinese hamster ovary (CHO) cell line was a generous gift from Dr. Pamela Reynolds (MRC, Harwell, UK). 35 mm glass bottom dishes were purchased from MatTech Corporation, USA.

HEK293 and HeLa cells were cultured in Eagle’s minimal essential medium (EMEM) (ATCC) containing 10% Foetal calf serum (FCS) and supplemented with 100 units/mL penicillin G sodium and 100 mg/mL streptomycin. CHO cells were cultured in DMEM/F12 supplemented with 10% FCS (Sigma) 50 units/mL penicillin G sodium, 50 mg/mL streptomycin, 2 mM L-glutamine (Invitrogen) at 37°C with 5% CO_2_ humidified air. Cells were either washed with x1 phosphate buffered saline (PBS) or maintained in full media prior to multiphoton confocal microscopy.

### Construction of EGFP-Rheb and DsRed-Rheb

The following reagents were obtained commercially: restriction enzymes (NEB), TOPO vector (Invitrogen), Miniprep and Midiprep kit (QIAGEN) and a Ligation kit (NEB). The Rheb cDNA clone contained in a pOTB7 vector (Gene service, IMAGE ID 3528583) and was PCR amplified using the forward primer containing *EcoR*I and the reverse primer containing the *Sal*I restriction enzyme site to clone EGFP at the N-terminus of Rheb. Also, pDsRedN1was PCR amplified using the forward primers containing *Age*I and the reverse primer containing *Bspe*I restriction enzyme site using pDsRedN1 as a template to create pDsRedC1 vector (Table 1). To clone EGFP at the N-terminus of Rheb, *EcoR*I and *Sal*I were used to digest a Rheb DNA fragment from the TOPO clone which was then gel purified and subcloned into *EcoR*I and *Sal*I sites of pEGFP-C1 to create EGFP-Rheb. To generate a DsRedC1 vector from DsRedN1, *Age*I and *Bspe*I were used to digest DsRedN1 DNA fragment from the TOPO clone which was subcloned into the *Age*I and *Bspe*I sites of pEGFPC1 to obtain DsRedC1. One positive clone was selected for further subcloning of DsRed-Rheb. The *EcoR*I and *Sal*I cut Rheb DNA fragments from the TOPO clone was subcloned into *EcoR*I and *Sal*I sites of newly generated DsRedC1 to produce DsRedC1-Rheb.

**Table 1:** Primers used for PCR amplification to construct EGFP-Rheb and DsRed-Rheb (5' – 3' direction; restriction sites underlined)

Rheb_EcoRI_FP	TGA ATTC GC CGC AGT CCA AGT CCC GG
Rheb_SalI_RP	GGG GTC GAC TCA CAT CAC CGA GCA TGA AGA C
DsRed(m)_AgeI_FP	AAC CGG TCG CCA CCA TGG AC
DsRed(m)_BspeI_RP	GGT CCG GA C TGG GAG CCG GAG TGG CG

### Construction of EGFP cloned to the C- and N-terminus of mTOR

The N-terminal tagged (EGFP-mTOR) and C-terminal tagged (mTOR-EGFP) vectors were constructed using mTOR cDNA contained in a pCMV6-XL4 vector. The cloning strategy to tag EGFP at the N and C-terminus of mTOR involved insertion of *Hind*III and *Sal*I restriction enzyme sites on both ends of mTOR using PCR amplification of the entire fragment of mTOR DNA. The Fermenta’s Long PCR Enzyme Mix (Ferments, UK) was used for PCR amplification with high fidelity DNA polymerase with proof-reading activity. The PCR amplification was performed by designing 4 primers (2 for each cloning) using the mTOR-pCMV6-XL4 vector as a template (Table 2). The cloning strategy involved three subcloning steps; the PCR product (7.6 Kb) (for EGFP-mTOR and mTOR-EGFP) was cloned into the PCR II TOPO vector and both ends of the amplified fragments sequenced, the DNA fragment from the original mTOR DNA fragment of the pCMV6-XL4 vector was replaced using *Nhe*I and *BspE*I restriction enzyme sites and the digested TOPO vector cloned into the *HindIII* and *SalI* sites of pEGFPC1 and pEGFPN1. Restriction digestion analysis, sequencing and Western blotting confirmed that the fluorescently cloned proteins were correct.

**Table 2:** Primers used for PCR amplification to construct EGFP-mTOR and mTOR-EGFP (5' – 3' direction; restriction sites underlined)

1_HindIII_FP	GGG AAA TAC GTA AAG CTT AAG ATG GGG CTT GGA ACC GG
4_SalI_RP	GGG CGG CCG CGT CGA CCC CCA GAA AGG GCA CCA GC
5_HindIII_FP	GGG AAA TAC GTA AAG CTT TAC TTG GAA CCG GAC CTG C
8_SalI_RP	GGG GTC GAC TTA CCA GAA AGG GCA CCA GC

### Construction of the DsRed-Raptor vector

The DsRed fluorescent protein was tagged to the N-terminal of raptor to construct a DsRed-Raptor vector. The DsRed-Raptor construct was generated from the haemagglutinin (HA) tagged raptor contained in a pRK5 expression vector. The cloning strategy to generate the DsRed-Raptor construct involved PCR amplification of a DsRed-PCRII TOPO vector (generated as above for DsRedC1 cloning) using the forward primer containing *EcoR*I and the reverse primer containing a *Sal*I restriction enzyme site (Table 3). The gel purified PCR product of DsRed was subcloned into a PCR-II TOPO vector. The *EcoR*I and *Sal*I digested DsRed DNA fragment from the TOPO clone was gel purified and subcloned into *EcoR*I and *Sal*I sites of a pRK5 expression vector containing raptor to generate a DsRed-Raptor vector.

**Table 3:** Primers used for PCR amplification to construct DsRed-Raptor (5' – 3' direction; restriction sites underlined)

DsRed(m)_EcoRI_FP	GGA ATT CAT GGA CAA CAC CGA GGA CG
DsRed(m)_SalI_RP	GGG GTC GAC CCC TGG GAG CCG GAG TGG CGG G

### Confirmation of fluorescently tagged Rheb, raptor and mTOR Proteins

Gel purified PCR products of Rheb and DsRed were subcloned into PCR-II TOPO vector (TA). The PCR products in TOPO vector were confirmed by restriction digestion analysis and sequencing (Gene service, Oxford). The restriction analysis of EGFP-Rheb and DsRed-Rheb was carried out by *Age*I restriction enzyme. Similarly cloning of DsRed-raptor vector was confirmed by restriction analysis by *EcoR*I and *Sal*I restriction enzyme.

The two mTOR constructs, EGFP-mTOR in which EGFP is tagged to the N-terminal of mTOR and mTOR-EGFP which contains EGFP at the C-terminal of mTOR were made. Initially the PCR product amplified for the cloning was confirmed for the correct sequence by cloning the PCR product in to PCR-II-TOPO vector, the cloning was verified by restriction digestion analysis and sequencing of both ends of PCR products. Using the correct clones, the middle fragment of PCR product in TOPO vector was replaced with a DNA fragment from the original mTOR vector following cutting of the whole fragment of mTOR from TOPO vector using *Hind*III and *Sal*I restriction enzyme sites and then cloned into pEGFPC1 and pEGFPN1 vector to get N-terminal and C-terminal tagged EGFP of the mTOR respectively. These final constructs were confirmed by restriction enzyme digestion by *BamH*I (see Tables 1, 2, 3 for primer details).

Rheb undergoes post-translational lipid modification by farnesylation at its C-terminal CAAX motif. Farnesylation is important for Rheb localization to endomembrane and activation of the mTOR pathway. Thus, to avoid any changes at the C-terminus of Rheb, EGFP and DsRed were located at the N-terminus of Rheb. In our current studies, the rather large and difficult mTOR has been successfully tagged with EGFP at either terminus and discussed below.

### Immunoblots of EGFP-Rheb, EGFP-mTOR and DsRed-raptor and an antibody to phosphorylated S6-kinase in HEK293 cells

Following 24 h or 48 h of transfection, HEK293 cells were detached by trypsinization and collected by centrifugation. Pellets were resuspended in ice-cold buffer (50mM Tris HCl pH 7.5, 350 mM NaCl, 1mM MgCl_2_), 0.5 mMEDTA, 1 mM EGTA, with protease inhibitor cocktail (Sigma) and NP40 1%) and lysates were subjected to Western blot analysis using specific antibodies to Rheb (Santa Cruz Biotechnology), and mTOR, raptor and phosphorylated S6-kinase (thr389) (Cell Signalling Technology, Inc.) as well as GAPDH to ensure loading onto gel lanes was the same. The data show that all three constructs were expressed, that they did not affect the level of endogenous/expressed levels of each other. The functionality of each of the tagged constructs was examined by probing the gels with an antibody to phosphorylated S6-kinase.

### Cell transfection

2x10^5^ cells were plated in 35 mm glass bottom dishes for 24h. Cells were transiently transfected with 0.5 μg of DNA using Fugene HD (Roche, UK) transfection reagent. Cells were then examined after 24-48h of transfection. Exponentially-growing cells were imaged under confocal and multiphoton excitation microscopy.

### Immunohistochemical staining

Cells were grown on 35 mm glass-bottom dishes at 2x10^5^ cells/dish and transfected with EGFP-Rheb for 24h. Following the transfection period the cells were fixed for 30 min with 4% (v/v) paraformaldehyde in PBS warmed to 37°C. Cells were washed three time and permeabilised with 0.2% Triton X-100 in PBS for 10 min followed by a further three washes with PBS. The cells were blocked for one hour in blocking buffer (1% BSA in PBS) and incubated with primary antibody (Rheb (C-19), Santa Cruz Biotech) in blocking buffer for 1h at room temperature, rinsed three times with PBS and incubated with fluorescently labelled secondary antibody (donkey anti-goat IgG-TR:sc-2783, Santa Cruz Biotech) (diluted in blocking buffer 1:200) for 1h at room temperature in the dark. The cells were then imaged with a Nikon TE2000U inverted microscope attached to a confocal microscope (see below for details).

### Co-localization stains

EGFP-Rheb transfected HEK293 and HeLa cells were stained with BODIPY TR C5 ceramide, ER Tracker Red (BODIPY TR glibenclamide) and Mitotracker red (all from Molecular Probes, Invitrogen) for live cell imaging. For Golgi staining, cells were washed twice with Hank's Buffered Salt Solution (HBSS), 5 μM BODIPY TR C5 ceramide in HBSS was then added and the cells incubated at 4°C for 30 min. Samples were washed several times with ice cold medium and incubated in fresh medium for 30 min at 37°C before processing for imaging. For ER Tracker staining pre-warmed HBSS with 1μM ER Tracker red stain was added to cells for 30 min at 37°C. For Mitotracker red staining, cells were incubated with Optimem reduced serum medium containing 100 nM of Mitotracker red stain for 30 min at 37°C. The staining solution was removed and the cells rinsed once with 1 ml of warm PBS prior to addition of 2 ml of pre-warmed fresh growth medium.

### Amino acid starvation

Serum starvation and amino acid re-stimulation experiments were performed by plating the cells into glass bottom dishes and transfected, as described above, followed by incubation for 24h. Cells were washed in PBS and maintained in culture medium containing penicillin/streptomycin and L-glutamine without FCS at 37°C and 5% CO_2_ in humidified air overnight (16-18h) until analysis. During amino acid starvation experiments, cells were washed once with 2 mL of warm (37°C) D-PBS. Followed by addition of 2ml of D-PBS and incubated at 37°C and 5% CO_2_ in humidified air for 1h. Amino acid-starved cells were stimulated using either 50x amino acid mixture (Sigma Aldrich, UK) or 10x amino acid mixture.

### Confocal and two-photon induced fluorescence time-correlated single photon counting image data acquisition

Confocal and multiphoton images of the fluorescent protein expression were collected using an inverted Nikon TE2000-U microscope attached to a Nikon C1 scanning unit with a GFP (488 nm excitation) or DsRed (543 nm excitation) filter set. Cells were selected for analysis on the basis of equal expression for all the protein constructs to ensure comparability between different interactions. All images were obtained using a 60× water immersion objective with a N.A. of 1.2. The data/images from the red and green channels were used to confirm at least an equal expression (or excess of the red fluorescence) prior to FLIM data collection. Fluorescence lifetime images were obtained using a two-photon-microscopy apparatus, which has an external x, y galvanometer scanning system (GSI Lumonics) {18}. Laser light at a wavelength of 920 ± 5 nm was obtained from a titanium sapphire laser (Mira, Coherent) producing 180 fs pulses at 75 MHz pumped by a frequency doubled vanadate laser (Coherent Lasers). Fluorescence emission was collected without descanning, by-passing the scanning system, and passed through a bandpass filter (BG39, Comar). The scan was operated in the normal mode and line, frame and pixel clock signals were generated and synchronized with an external fast micro-channel plate photomultiplier tube (Hamamatsu R3809U) used as the detector. These were linked *via* a time-correlated single photon counting (time-correlated single photon counting) TCSPC PC module SPC830 (Becker and Hickl). The set up provided instrument quantum efficiencies of more than 50% with single photon detection capabilities. The non-descan method also allows increased signal detection over the one-photon method. Data of *in vitro* one and two photon fluorescence and emission lifetime studies of these TCSPC lifetime imaging micrographs were analysed using SPC Image analysis software (Becker and Hickl, Germany). The lifetime distribution histograms presented are determined from calculations at each pixel in the FLIM image at each individual single pixel x, y such that I (*t*_*i*_*, x*_*j*_*, y*_*j*_) is the fluorescence intensity at time t_i_ in pixel xy_j_. The decay must be convolved with the instrumental response function (IRF). Thus each recording of the fluorescence decay in a pixel can be considered as a separate experiment. The accuracy of the decay fitting is characterized by a χ^2^ close to 1. We have determined our instrument response function to be 50 ± 10 ps, so that this can be ignored in our data analysis since the changes we report (>100 ps) are significantly larger. The quoted lifetime values are derived from summing up and averaging all the pixel values (generally 128 x 128) i.e. 16348 individual values to derive the pictorial histogram and associated error bars from this and the repeat of at least 3 independent experiments.

## Abbreviations

FRET: Fluorescence Resonance Energy Transfer; FLIM: Fluorescence Lifetime Imaging Microscopy; EGFP: Enhanced Green Fluorescent Protein; mTORC1: Mammalian Target Of Rapamycin Complex 1; mTOR: Mammalian Target Of Rapamycin; TCSPC: Time-Correlated Single Photon Counting.

## Competing interests

The authors declare that they have no competing interests.

## Authors’ contributions

RBY PB AWP VI CGP RAA JPO AJ CDS SWB participated in research design. RBY VI CGP AJ CDS SWB conducted experiments. RBY VI CGP CDS SWB performed data analysis. RBY AWP VI CGP CDS SWB wrote or contributed to the writing of the manuscript. All authors read and approved the final manuscript.
